# Perceptions of complementary and alternative medicine among cardiac patients in South Trinidad: a qualitative study

**DOI:** 10.1186/s12906-015-0577-8

**Published:** 2015-03-31

**Authors:** Mandreker Bahall, Mark Edwards

**Affiliations:** Arthur Lok Jack Graduate School of Business, Max Richards Drive, Champ Fleurs, Mount Hope, Trinidad; Faculty of Medicine, University of the West Indies, St. Augustine, Trinidad and Tobago; University of Liverpool, London, UK; University of Roehampton, London, UK

**Keywords:** Complementary and alternative medicine, Patient perception, Cardiac patient, Integrative medicine, Holistic medicine

## Abstract

**Background:**

Complementary and alternative medicine (CAM) has been practiced for centuries owing to the absence or limited availability of conventional medicine. CAM has persisted globally with over USD34 billion spent annually, despite modernization, globalization, technological advancement, and limited supportive evidence. The present qualitative study explores the perception of CAM among cardiac patients with respect to rationale, perceived outcomes, influences, and public health concerns.

**Methods:**

This study used a qualitative, interpretative approach. Twelve cardiac disease patients were recruited from private clinics in South Trinidad and interviewed. The study obtained ethical approval, and all participants provided written consent. The semi-structured interviews were digitally recorded, transcribed, and thematically analyzed. Participants with poor cognitive function, difficulty speaking, and those not understandable owing to language barriers were excluded.

**Results:**

CAM use was based largely on patient perception regardless of the clinical reality. The perceived mode of action and its natural character was responsible for the therapeutic outcomes and uses. Participants reported that CAM provided holistic care, improved the quality of life, overcame the limitations of conventional medicine, satisfied their increased expectation for comprehensive care, and prevented or counteracted adverse effects caused by conventional medicine. Participants reported a lack of scientific information on CAM and stated that policy makers should assist patients through increased research, public health education, and improved integration of CAM and conventional medicine.

**Conclusions:**

The participants’ use of CAM was largely based on perception. CAM was thought to improve therapeutic outcomes, provide holistic care, decrease or prevent complications from conventional medicine, and improve quality of life. Participants acknowledged that they may be ill-informed about the basic concepts or actions of CAM. They urged policymakers to create an environment that assists the public and health care providers in promoting safe and effective CAM practice.

## Background

The use of complementary and alternative medicine (CAM) is increasing worldwide. Reportedly, up to 61% of cardiac patients with coronary artery disease and patients at risk of arteriosclerosis use CAM [1]. Among patients at risk of and diagnosed with cardiovascular disease, approximately 26 to 42% use supplements as a component of therapy or prevention [2]. At least 82.5% of cardiac patients used some form of CAM according to a study conducted by the Mayo Outpatient Cardiac Clinic [3]. Global CAM use ranges from 9% to 65%, and its use by cardiac patients in particular ranges from 4% to 61% [4]. Barnett [5] reported that 5 million patients are examined by a CAM practitioner annually in the UK, and according to Tachjian [6], 15 million patients annually see a CAM practitioner in the USA. The most commonly employed treatments are relaxation techniques, herbal medicine, massage, chiropractic treatment, and meditation [7].

CAM use among cardiac patients in Trinidad has not been studied, though unpublished data from the author suggests that as many as 56% of cardiac patients may use CAM. However, there is a lack of understanding about how patients in Trinidad and Tobago perceive CAM, its techniques, and indications for various cardiac conditions. Trinidad and Tobago is a small twin island state with a population of 1.3 million people. It is a cosmopolitan society with a long history of CAM and traditional medicine. The public health care system is free but inadequate. Cardiovascular disease accounts for 33.7% of all deaths in the Americas, with Guyana and Trinidad and Tobago showing the highest mortality rates in the region [8]. CAM has long been traditionally practiced in Trinidad [9] and for decades has been the major and sometimes the only medical treatment available owing to the lack of modern health care [10]. A complete understanding of patient perceptions is particularly critical owing to the risks of herb–herb and herb–drug interactions, and direct herbal toxicity. The use of CAM continues unchecked, and guidelines and evidence supporting its claims are lacking [11]; it continues to be used even when sound reason and logic is absent [6]. Patients gravitate to unregulated CAM practitioners who have questionable safety practices and efficacy, and may even cause harm [12]. In contrast to these concerns, several CAM studies have reported positive experiences among patients. According to a study in Flemish adults aged 18 years and over, 70% of respondents did not consider CAM as a public health threat [13]. CAM use may also influence cardiac medicine practice. Hassen et al. [14] surmised that CAM may potentially replace and influence patient’s compliance and clinical outcomes in patients receiving conventional therapy.

In order to mitigate the potential negative consequences of CAM, the World Health Organization (WHO) Traditional Medicine Strategy of 2002–2005 has listed four public health concerns for CAM, namely CAM policy; patient safety, therapeutic efficacy, and quality; patient access; and rational therapeutic use [15]. Salomonsen et al. [16] recommended that traditional health care providers cooperate with CAM practitioners owing to the increased acceptance by referral institutions on the possible effectiveness of certain CAM therapies. With its expanding role in public health, CAM usage, including among conventional doctors, must be re-evaluated in order to encourage more rational, safe, and effective use [17]. Evidence supporting CAM has encouraged a paradigm shift by The National Centre for Complementary and Alternative Medicine (NCCAM) and caused the organization to incorporate relevant and validated CAM and conventional medicine into a single discipline known as integrative medicine [18]. According to a former Chief Medical Officer of Trinidad and Tobago, studies should prioritize public safety and ensure that CAM claims can withstand scientific scrutiny [19]. An understanding of patient perception will improve public health through increased empowerment, community awareness, and improved understanding of CAM by encouraging appropriate, safe, and effective treatment choices by patients and health care providers [20]. This study explores the perceptions of CAM by cardiac patients using in-depth interviews to evaluate the perceived rationale, therapeutic outcomes, challenges, influences, and public health concerns. This will help health care providers to understand CAM practice to improve decision making at the policy level as well as at the customer level.

## Methods

A perceptive interpretivist approach was used as this technique is able to explore the participant’s perception of their personal and social world [21]. This approach allows us to understand the thinking and behavior of patients as perception rather than objective evidence may actually determine health-related behavior. Other qualitative methods such as ethnography and field research, which require participant observation, and grounded theory, which is a complex iterative process, were deemed less appropriate. In-depth interviews were used because participant observation (observing participants in their daily lives) and focus groups (obtaining broad overviews or cultural norms) were ill-suited to the study objectives.

All cardiac patients treated at private clinics in South Trinidad were eligible. However, the study population came from three private clinics. Interviews were conducted on 12 cardiac patients recruited from them to participate in the survey (Table 1). Ethical approval was obtained from the Ethics committee of the Trinidad and Tobago Ministry of Health in November 2012, and all participants provided written informed consent after the purpose and nature of the study was disclosed. Participants were assured that the solicited information would not affect their treatment, and they were free to terminate the interview at any time. Participants were included according to the following criteria: African, East Indian descent, or mixed ethnicity; male or female; and adults according to three age groups (<45 years, 45 to 65 years, and >65 years). Participants with poor cognitive function (confused, disoriented, bewildered, or inappropriate behavior) or who had difficulty communicating in English were excluded.

**Table 1:** **Participants’ health status/setting**

**Date of interview**	**Location of interview**	**Identity no.**	**Interviewee profile**
20/02/12	Medical Centre	P01	A 53-year-old man of East Indian descent lives with his wife and children in Barrackpore. He is a known hypertension and hypercholesterolemia patient with ischemic heart disease for 3 years. He had a heart attack in 2012 and underwent open heart surgery in 2012.
20/02/12	Medical Centre	P02	A 63-year-old man of East Indian descent lives with his wife in Gaspairllo. He is known to have diabetes, hypertension, and hypercholesterolemia. He had a heart attack in 2010.
30/01/13	Medical Centre	P03	A 78-year-old female housewife of mixed descent is a known diabetes and hypertension patient and lives with her family in Marabella. She has been experiencing recurrent palpitation for the past 3 years.
03/02/13	Home	P04	A 53-year-old unmarried man (***Participant***) of African descent from Couva with a full time job has had coronary artery disease for about 2 years and experienced a heart attack in 2011.
03/02/13	Home	P05	A 71-year-old man (***Participant***) from Chaguanas, who lives alone has had severe coronary artery disease and recurrent angina for at least 2 years.
04/02/13	Medical Centre	P06	A 70-year-old woman (***Participant***) of East Indian descent, who lives in Penal with her granddaughter, is a known diabetes, hypertension, and hypercholesterolemia patient with ischemic heart disease for about 2 years.
04/02/13	Medical Centre	P07	A 63-year-old woman (***Participant***) of mixed descent from Barrackpore, who lives with her husband is a known diabetes and hypertension patient with ischemic heart disease for the past 4 years. She underwent an open heart surgery in September 2011.
05/02/13	Medical Centre	P08	A 69-year-old woman (***Participant***) of Indian descent, who lives in Barackpore, is a known diabetes, hypertension, hypercholesterolemia patient with ischemic heart disease for more than 6 years.
05/02/13	Medical Centre	P09	A 78-year-old widow (***Participant***) of East Indian descent from Princess Town who lives with her granddaughter is a known hypertension patient with severe aortic valve disease. She and underwent valve replacement in November 2012.
05/02/13	Medical Centre	P10	A 67-year-old woman female vendor of East Indian descent is a widow who lives with her children in Freeport. She is diabetic, hypertensive, and asthmatic and has had ischemic dilated cardiomyopathy (weak swollen heart) for the past 2 years.
30/01/13	Medical Centre	P11	A 68-year-old male of East Indian descent is from Gasparillo and lives with his wife and children. He is a known diabetes, hypertension, and hypercholesterolemia patient and has had severe coronary artery disease for about 2 years. He underwent open heart surgery in May 2012.
14/02/13	Medical Centre	P12	A 29-year-old single man of African descent works part time and lives in Guapo. He is hypertensive and has had dilated cardiomyopathy (weak heart) for about 2 years.

The semi-structured in-depth interviews lasted approximately 45 to 60 minutes. The interviews were conducted using an interview guide comprising numerous main questions and sub-questions covering the main themes of the subject. Participants were questioned on their understanding of CAM, reasons for using CAM, the context of CAM usage, influences on their CAM usage, challenges encountered, overall experience, complications experienced, perceived clinical outcome, and future implications. Subtopics covered these categories as follows: Knowledge: past usage, known CAM treatments, and previously experienced treatment; Reasons: cost, efficacy, clinical outcomes, coercion, and peer pressure; Context: social, cultural, belief systems, and background; Influences: personality, peer pressure, and tradition; Challenges: adverse effects, transparency (or lack thereof), and evidence-based information; Complications: liver failure, hemorrhage, hypoglycemia, kidney failure, and seizures; public health concerns; and future implications.

In this study, CAM was defined as any treatment other than that prescribed by a medical professional [22] as described by NCCAM at the National Institutes of Health. NCCAM defines CAM as “a group of diverse medical and health care systems, practices, and products that are not generally considered part of conventional medicine”, including herbs, dietary supplements, meditation, biofeedback, hypnosis, acupuncture, Ayurveda, homeopathy, naturopathy, Chinese medicine, chiropractic medicine, massage, Tai chi, yoga, electromagnetic therapy, kinesiology, Reiki, and Qigong [18].

Initially, two interviews were conducted on December 28, 2012 as a pilot study in order to detect any problems or challenges associated with patient recruitment, ensure that the study was conducted in a culturally appropriate manner, and modify the interview guide if needed [23]. The information obtained in the two pilot interviews was adequate and was included in the general analysis. The interviews were recorded digitally and transcribed in English by the researcher, typed by a typist, and safely secured on a computer. The remaining 10 interviews were conducted from January 3–14, 2013 over six sessions, with one to three interviews per session. The digital records were accessible to the researcher and assistant via a password and will be disposed of electronically or by shredding after 5 years. To ensure privacy, patients were anonymized and identified by a unique number (P01 to P12).

The interview transcripts were reviewed for omissions and grammatical errors. Interviews were subjected to an inductive thematic analysis including coding and identification of common or recurrent themes using a comparative analytic process of each participant’s views [24]. Codes common among the participants were identified, and those belonging to a particular theme were color coded. The color-coded themes were coalesced into a single document, and the themes were mapped to show correlations among the participants according to the CAM type, knowledge, mode of action, properties, public health concerns, and challenges. The data were further analyzed to assess each participant’s behavior based on the unique codes [24]. Both the researcher and participants shared a similar cultural background, spoke English and the Trinidad dialect, did not have any communication barriers, and were exposed to CAM practice either directly or indirectly. The participants lived in South Trinidad, had low to middle income, and were delighted to share their experiences with CAM. Interviews were conducted privately in a comfortable and spacious office with few distractions.

## Results

### Participant characteristics

The 12 participants included Africans (2), East Indian descent (8) and mixed ethnicity (2) patients. Men comprised 40% of the group, and various age groups were represented. Trinidad and Tobago citizens are predominantly African (34.2%), East Indian descent (35.4%), and mixed (22.8%) ethnicities [25]. In South Trinidad, the ratio of East Indian descent to Africans is 2.2:1, and ischemic heart disease is more common among people of East Indian descent. All participants were previously diagnosed with heart disease including coronary artery disease (84%), valvular heart disease (8%), and cardiac arrhythmia (8%).

### General types and trends in CAM treatment

Patients used a number of herbal products including: goji berry (*Lycium barbarum L*), spearmint (*Mentha spicata*), ginger root (*Zingiber officinale*), saffron (turmeric), garlic (*Allium sativum*), orange peel, apple cider, kanchan water, Neem (*Azadirachta indica*), tulsie (holy basil used by Hindus), goji juice (lyceum fruit), Pica bush (*Sambucus canadensis L*), bandanya (fitweed, recau, shado beni), red stinger nettle *(Urtica urens)*, Carailli (*Momordica charantia L*.), Zebapeek (*Neurolaena lobata R. Br*.), and soursop leaf (*Annona muricata*). Other CAM therapies included jarre (special Hindu prayers), chelation therapy (administration of chelating agents to bind metals, minerals, and other molecules), and healing prayers. The participants used CAM to address conditions not alleviated by conventional medicine, treat adverse effects caused by conventional treatments, provide holistic care, create a feeling of wellness, and generate a sense of control. CAM usage was encouraged in part by the testimonies of others and decades of traditional medicine in Trinidad. However, usage was primarily determined and encouraged by each participant’s perception of CAM, which is further explored in the following analysis. The perceived characteristics, mode of action, rationale, roles, and clinical outcomes explains why and how people use CAM and conventional medicine both in isolation and in combination.

### General perception of CAM

The participants defined CAM as “*a herbal medicine or something you use from your kitchen*” (P03). Herbs were further characterized as containing *“no preservative nothing to damage you or anything so it is good to take”* (P10), and herbal products were thought *“[to] not have side effects” (P05)* and “*could work with anything*” (P02). Participants typically linked CAM with purity and “*natural*” (P01, P06) and believed that CAM had universal applications without any potential for harm. The perceived safety profile was reinforced by a long history of CAM use in the form of traditional Trinidadian medicine that has been passed down over centuries and is perceived as safe and acceptable. One participant stated that “*from the time I was young, I tell you that my grandmother uses to boil, pick up the bush and boil it, she would mix it with honey, sometime she might put a little bit of Vicks or a little bit of olive oil too and mix it up–for the cold/bronchitis”* (P01). Various types of CAM as earlier mentioned were identified by patients to treat cardiac diseases such as coronary artery disease, thrombosis (blood clots), cardiac arrhythmias (abnormal heart rate) and dilated cardiomyopaties (weak dilated heart).

### Roles of CAM and conventional medicine

Participants perceived that CAM and conventional medicine served different roles but complemented each other. CAM was thought to be effective in *“cleaning the arteries little by little*” (P02), while conventional medicine such as trimetadizine (Vastarel) helped “*to heal the muscles of the heart*” (P02). One participant asserted that as long as the serum cholesterol concentration was low, dietary restriction was unnecessary as CAM “*protects the system from getting probably contaminated or getting disease and so on or getting any ailment*” (P01). The participants described the role of CAM with great clarity. Participants reported that a lack of knowledge on the risks and complications of conventional medicine was responsible for unhealthy lifestyle choices and the use of conventional medicine. As asserted by one participant, *“My people perish for the lack of knowledge; for [conventional medicine] is cancerous. Why would I put that [conventional medications] in my body?”* This participant continued that *“you could eat the sweetest orange, banana, the sweetest fig, mango, cherry, whatever, it could be how high in sugar, your body would process that because your body was designed to process that”* (P04). According to him, conventional medicine was thought to have a “*million side effects yet it is given to heal patients*” (P04)*.* By contrast, CAM usage was glorified as a form of natural healing. Participants claimed that *“whatever side effects you get from the conventional medicine, the organic medicine might take away the side effect too”* (P05). Participants asserted that CAM did not have adverse effects, and according to one participant, he “*doesn’t think CAM can be dangerous*” (P01). Notably, one patient admitted that CAM has the potential *“to cause death”* (P02).

### Benefits of CAM

Patients experienced both clinical and non clinical outcomes. It is difficult to determine whether the clinical outcomes in participants using CAM was due to the treatment itself, a placebo effect, or another confounding factor. Regardless, participants credited CAM for their positive clinical outcomes and boasted of its symptom relief and their improved quality of life. The participants reported that there were no significant negative outcomes associated with CAM usage. Cardiac patients who previously used CAM reported significant improvements in clinical symptoms including improved breathing, stronger appetite, and decreased chest pain. Several elderly participants with a history of myocardial infarction and poor cardiac function reported an improved quality of life. The participants reported feeling more well, stronger, healthier, livelier, and more energized (P01, P12), and having increased stamina (P12). Some participants reported feeling cured and that they could live “*a long healthy life*”; others reportedly *“don’t get any chest pain at all”* (P05). The participants also reported that CAM encouraged improvement in their general wellbeing through lifestyle changes. One patient stated that *“You can’t go back to your old habit, you have to change it, so that in itself is a cure because you not going back to eat so much*” (P07), and another reported that “*I used to feel a lot of weakness; I used to have to go for booster all the time but now I don’t have to get nothing … I am 68 years old and I must say I does feel like a little girl, I could still run up the step and I know it is these things that help me, because I not on any other heart medication”* (P11).

Patients had more “input and say” in CAM. Despite feeling insecure at times, participants reported feeling less subservient with CAM. They felt more in control of their treatment using CAM products as well as the choice of their CAM provider. Participants therefore wished to “*get as much information as they could on these products, those things, their side effects and how it works on the human body”* (P08). Such empowerment, many claimed would facilitate appropriate decision making.

### Influences on CAM usage

Positive testimonies were a major factor promoting CAM usage. Glowing recommendations and tributes made CAM usage irresistible. Participants boasted of increased longevity in elderly people who used traditional medicine and marveled at the improved physical appearance, fitness, and psychological wellbeing of those who used CAM: *“look at all those who live hundreds of years, they use herbal medicine. So that is what convinces me that it is working”* (P05). Participants were also influenced by scientifically unreliable sources such as the internet, friends, family and lay people for information on CAM.

### Combined use of CAM and conventional therapy

The use of CAM alone and in combination with conventional medicine was decided according to perception. The perceived safety of single or combination therapy varied among the participants. While participants felt that CAM alone had definite benefits without adverse effects, they were concerned over the possibility of adverse effects with combination therapy. Several participants preferred to use one treatment at a time, asserting that *“Conventional and herbal does not mix you know”* (P08) and *“because I don’t really know if it has the two together if it would do something wrong*” (P06). However, half of the respondents felt that it was necessary to use both therapies in combination and did not want to risk declining conventional medicine. One participant reported that combined therapy had a synergistic effect because CAM was safe: *“they will fight together*” (P10). The lack of knowledge and the possible loss of benefit from conventional medication posed a dilemma, which was addressed by using both therapies, according to one participant (P01). One patient asserted that *“it [CAM] doesn’t hamper with the use of the other medication which I taking and that is how I decided to try it out and the results is good so far” (*P12). Notably, several participants reported discontinuing CAM intermittently and continuing with conventional therapy alone because they did not wish to incur the risks of using CAM at the expense of conventional treatment. Occasionally, conventional therapy was discontinued to allow use of CAM alone.

### Challenges: access to CAM and integration with CM

Participants were generally convinced of the value of CAM but expressed two major concerns: the inaccessibility of certain types of CAM and providers; and the lack of support, knowledge, and integration by conventional health care providers. Most CAM was based on simple herbal and juice preparations purchased in local stores or prepared at home. Some conventional medications such as clopidogrel (Plavix) and rosuvastatin calcium (Crestor) may be unavailable through the free public health system and can be prohibitively expensive at private pharmacies. Rather than declining therapy entirely, some participants sought simple inexpensive or free forms of CAM. According to one participant, the *“prices [of conventional medications] in the market are real, real ridiculous*”, and local CAM providers *“would treat you and give you the best treatment and would allow you to leave without taking a cent*” (P04)*.* However, a few of the treatments purported to eliminate arterial obstruction require imported herbal products. While some participants were willing to make sacrifices to purchase CAM, others found CAM to be expensive (P02, P07), especially when imported or foreign products were required (P05). According to one participant, “*you cannot just go in the drug store and buy it, you must order from the States”* (P02). Another challenge was the discipline and time required to prepare CAM products locally such as herbal juices.

Participants reported a willingness to disclose their concerns of CAM with conventional medicine practitioners. However, participants reported that conventional practitioners “*don’t believe in what you using CAM for*” (P07) and that “*doctors do not want to know about herbal medicine*” (P05). Therefore, participants did not receive support from health care providers. This poses a major challenge because of the possible consequences of single or combined usage of CAM. Many felt that CAM should be integrated with CM.

### Role of government and health care providers

The participants acknowledged their lack of objective information on CAM, which if available, would allow them to make informed choices. They believed that health authorities should conduct additional research to ensure safe and evidence-based practices. One participant stated that *“People, who are doing their traditional medicine, should do a little more research and make it more accessible to the public” (*P04), and another similarly asserted that health care providers must inform the public on *“how to mix it, and how to take it”* and that governments have *“to set their standard” (P05).* Participants expressed a desire for the government to play a role in training doctors and providing evidence-based information on CAM therapy which should be integrated with CM.

## Discussion

The use of CAM is based largely on patient perception regardless of the clinical reality. The perceived characteristics, rationale, mode of action, and therapeutic outcomes of CAM influence its use. With scientific advancements and an improved understanding of the pathophysiology of many diseases, one would expect patients to welcome, accept, and rely on evidence-based guidelines to manage cardiac disease. On the contrary, perception, despite its unscientific nature, continues to influence the use of CAM alone or in combination. This has led to patient complications and a major public health problem. The present study examined the subjective experiences and interpretations of each participant in order to gain insight on their use of CAM according to previously described phenomenological theory [26]. The participants in this study primarily used CAM in the form of herbal remedies, a trend that was also reported by Tachijian [6].

Patients seek to alleviate their cardiac disease, improve their quality of life and wellness, and potentially cure the disease. The desire for more holistic and natural approaches and the greater philosophical congruence between CAM and cultural practices (pull factors) is high [27]. Grossman [28] similarly reported an increased demand and expectation by patients for more holistic and comprehensive care. In a qualitative study conducted in the USA, Greene [29] reported that patients experienced empowerment, increased hope, and spiritual benefits after receiving CAM treatment. However, the usage of CAM depends heavily on local socioeconomic characteristics and cultural practices of the particular society [30]. These factors also dictate the high patient satisfaction despite the limited understanding of CAM [30].

Patients are encouraged to use CAM because of dissatisfaction with and adverse effects stemming from conventional therapies (push factors). The possible adverse effects listed in drug inserts may dissuade patients against conventional medications and drive them towards CAM treatment. Many patients believe that prescription drugs cause more harm than other therapeutic options [31]. This perception greatly favors CAM, especially if there are no toxic effects, interactions, or interference with conventional treatment.

However, the claim to provide safe and effective treatment resulting from pull and push factors is not always true. Furthermore, there is the potential for herb toxicity and herb-drug interactions, and patients may even discontinue conventional treatment in favor of CAM. The risk of hemorrhage may increase when certain cardiac drugs such as aspirin, clopidogrel, and warfarin are taken together with ginkgo [32] or garlic (*Allium sativum* L) supplements [33], though this risk has not been found in other studies [34-36]. Many cardiac patients experience allergies, toxic reactions, mutagenic effects, and even death with CAM use [20,37]. Paul and Seaforth [38] found that the continued popularity of folk remedies in Trinidad potentially exposes people to dangerous toxins. Our present findings are consistent with those of White et al. [39], who found that patients perceived CAM as less harmful and conventional medicine as ineffective and with other reports in which patients perceived CAM as natural and safe [1] or cost-effective [40]. The attractiveness of CAM arises from its perceived ability to prevent and treat illness with minimal adverse effects while decreasing complications caused by conventional medications [41]. Despite the overall positive attitudes and acceptance of CAM by patients, conventional medicine was not generally condemned, although many patients felt that conventional therapies did not meet their therapeutic goals. This attitude likely indicates that patients do not completely trust CAM to perform all the roles served by conventional therapy. It therefore complements the role of conventional medicine. This dichotomous and complementary role encourages and expands the use of CAM. Patients are aware of the limitations of conventional medicine and therefore seek alternative treatments. This is well recognized for cardiac diseases especially with patients with a history of atherosclerosis where there no cure. Patients welcome and embrace CAM because it fills the void left by conventional medicine. CAM promises to alleviate and even prevent coronary artery blockage.

Participants justify their usage of CAM through its perceived mode of action as an anticoagulant and its perceived ability to relieve arterial obstructions and improve cardiac function. These attitudes suggest that patients do not understand the basics of cardiac disease. Corner et al. [42] found that some patients actually avoided CAM due to a lack of interest in CAM, satisfaction with conventional treatment, confidence in the doctor’s therapeutic recommendations, and a lack of information on the safety, cost, and availability of CAM. Some researchers found otherwise. CAM may be inappropriate and even toxic in some cases. Many CAM products have not been tested for toxicity.

Participants were also motivated by the perceived positive outcomes or benefits of CAM. Participants who reported feeling more energetic, active, and psychologically stronger were encouraged to continue CAM. Similarly, Kreitzer and Snyder [43] concluded that psychological factors such as stress, anxiety, anger, hostility, social isolation, and depression were relieved by lifestyle interventions such as diet, exercises, yoga, and group support, ultimately improving and even reversing coronary artery disease risk factors. In another study, positive outcomes including improved symptoms, better quality of life, disease cure, and an improved will to live significantly encouraged CAM use [44]. Participants reported dissatisfaction and a sense of hopelessness due to the inadequacy of conventional therapies. However, it is unclear whether a subjectively improved quality of life correlates with the quality of life measured comprehensively. In a study of cardiovascular disease and diabetic patients, Canaway and Manderson [45] found that patients reported an improved quality of life, but the quality of life as tested by a questionnaire worsened.

Many patients recognize the gross inadequacy of the health system in a multitude of areas including pharmacy, radiology, and cardiology services. Despite the inconsistent and sometimes inadequate medical care, patients typically assume a subservient role and follow the recommendations of conventional practitioners. Thus, health care providers essentially determine treatment. However, CAM users can select their treatment and CAM providers, sometimes at no cost. The perceived caring nature of CAM providers [5] contrasts with busy conventional medicine practitioners. While not explored in detail patients felt they had more control in deciding the type, route, and goals of their treatment. They expressed the desire to do more for themselves. According to Warren et al [46] patients report feelings of empowerment, autonomy, agency, and self-efficacy. Such feelings were also captured by Manderson and Cannaway [47]. While the management of cardiovascular disease is considered complex and too large a task to address with CM, alternative medicine practitioners provide patients with the individualized attention and care that is required to assist them with their health concerns [47].

In the present study, CAM usage was motivated by high expectations: push and pull factors, positive outcomes and the desire for greater control. It was influenced by friends, family, cultural tradition, apprehension to seeking medical care in conventional hospitals, and a personal preference for CAM; these factors were also identified by Mariam et al. [48], who found that patients were motivated to use CAM by recommendation from family and friends, family sanctions, perceived benefits, healer credibility and reservation with Western medicine. Trinidad and Tobago’s long history of traditional medicine and extended family support also encouraged the use of CAM in the present study. This trend was observed by Lee et al. [49], who found that CAM use was more likely in patients strongly supporting traditional theories of health, illness, and remedies and in those encouraged by family members.

The firm belief in the value of CAM in the face of strong evidence supporting conventional treatment is a cause for concern. The use of CAM prior to seeking medical attention can delay conventional therapy, which may compromise the quality cardiac care and lead to cardiac complications [50]. In patients with coronary artery disease, this delay can lead to myocardial infarction or even death. CAM use continues though some studies report a lack of perceived effectiveness [51]. In a study of patients with cardiovascular disease, Whayne [52] found that CAM treatment was worthless in some patients, and other patients suffered harm and a higher risk of negative outcomes with no therapeutic benefit, while the CAM practitioners benefited financially. Dangerous CAM practices continue, especially in patients receiving potentially toxic cardiovascular medication [53] and are a major public health concern.

Despite these risks, some CAM treatments have withstood scientific scrutiny and are prescribed by conventional practitioners as a form of integrative medicine. The present study highlights a number of public health concerns in CAM including misconceptions on the mode of action and indications, a lack of monitoring, unsubstantiated testimonies and advertisements, unrestricted and unregulated use, the lack of guidelines, and inaccurate information. CAM’s efficacy is also questionable and may compromise patient health by delaying effective treatment, encouraging the wrong treatment, and by interfering with conventional treatment. Despite these risks, our findings indicate that patients are willing to accept CAM based on perception. Unfortunately, CAM use is typically not reported to conventional practitioners because many patients feel that health professionals would be unable to accommodate their concerns and lack of knowledge on herbal remedies and other CAM types. These same factors were discussed previously in the Socio-Behavioral Model of Anderson, which explored the determinants for CAM use in a model for health service utilization [54].

With the paucity of reliable information [40], the participants felt that governments had a responsibility to provide scientific guidelines, educate health care providers, and empower patients to ensure effective CAM practice. Participants felt that all stakeholders, including the public and conventional practitioners, should receive sound scientific information to encourage safe and effective CAM practice. Integration of CAM and conventional medicine is needed [55]. Additional research is required investigating the determinants of CAM usage and interventions that can change patient perceptions and modify health behaviors.

### Study limitations

The sample frame came from three private cardiac clinics which may not be representative of cardiac patients. Another limitation is that the study did not include adequate number of participants from all ethnic subgroups. The present sample population included fewer Africans than in the general population. However, in South Trinidad where the study was conducted, descendants of East Indians outnumber Africans more than 2:1, and cardiac disease is more prevalent in the East Indian descendent population. The small sample size is also a concern, and a larger sample may have revealed greater variation in CAM perceptions and practices. The study also failed to compare perceptions of CAM between users and nonusers in the cardiac disease population. Potentially, because the survey was administered by a health professional, participants may have been unwilling to fully disclose their perceptions and use of CAM. To minimize this, patients were assured that their responses would not affect their medical treatment. The researcher conducting the interview was a health professional with a deep understanding of health issues concerning both conventional medicine and CAM. In order to minimize bias, he spent time engaging in reflexivity so that he can better represent the findings of patients [56]. Such information, nonetheless, provides detailed and in-depth understanding than objective filters as actual patient behavior is best explained through differing perspectives.

## Conclusions

CAM usage persists among cardiac patients due to perceptions of its characteristics, mode of action, therapeutic outcomes and expectations. CAM is perceived by patients to improve wellbeing and the quality of life, counteract the adverse effects and toxicity of conventional medications, and engender a sense of control over health decisions. Factors favoring CAM usage include family, friends, and other supporters, electronic and print media, CAM providers, and positive testimonies from other patients. Overall, patients are willing to use CAM but are unwilling to discontinue conventional medicine. The use of CAM with or without conventional therapy poses a major public health concern.
